# Lifestyle Behavior Changes and Associated Risk Factors During the COVID-19 Pandemic: Results from the Canadian COVIDiet Online Cohort Study

**DOI:** 10.2196/43786

**Published:** 2023-03-30

**Authors:** Anne-Julie Tessier, Audrey Moyen, Claire Lawson, Aviva Ilysse Rappaport, Hiba Yousif, Chloé Fleurent-Grégoire, Sophie Lalonde-Bester, Anne-Sophie Brazeau, Stéphanie Chevalier

**Affiliations:** 1 School of Human Nutrition Faculty of Agriculture and Environmental Sciences McGill University Sainte-Anne-de-Bellevue, QC Canada; 2 Research Institute of the McGill University Health Centre Montréal, QC Canada

**Keywords:** healthy lifestyle, behaviors, pandemic, COVID-19, public health, body image, gender, stress, risk factor, physical activity, public health, mental well-being, depression

## Abstract

**Background:**

The COVID-19 pandemic and related lockdowns have impacted lifestyle behaviors, including eating habits and physical activity; yet, few studies have identified the emerging patterns of such changes and associated risk factors.

**Objective:**

This study aims to identify the patterns of weight and lifestyle behavior changes, and the potential risk factors, resulting from the pandemic in Canadian adults.

**Methods:**

Analyses were conducted on 1609 adults (18-89 years old; n=1450, 90.1%, women; n=1316, 81.8%, White) of the Canadian COVIDiet study baseline data (May-December 2020). Self-reported current and prepandemic weight, physical activity, smoking status, perceived eating habits, alcohol intake, and sleep quality were collected through online questionnaires. Based on these 6 indicator variables, latent class analysis (LCA) was used to identify lifestyle behavior change patterns. Associations with potential risk factors, including age, gender, ethnicity, education, income, chronic diseases, body image perception, and changes in the stress level, living situation, and work arrangement, were examined with logistic regressions.

**Results:**

Participants’ mean BMI was 26.1 (SD 6.3) kg/m^2^. Of the 1609 participants, 980 (60.9%) had a bachelor’s degree or above. Since the pandemic, 563 (35%) had decreased income and 788 (49%) changed their work arrangement. Most participants reported unchanged weight, sleep quality, physical activity level, and smoking and alcohol consumption, yet 708 (44%) reported a perceived decrease in eating habit quality. From LCA, 2 classes of lifestyle behavior change emerged: healthy and less healthy (probability: 0.605 and 0.395, respectively; Bayesian information criterion [BIC]=15574, entropy=4.8). The healthy lifestyle behavior change group more frequently reported unchanged weight, sleep quality, smoking and alcohol intake, unchanged/improved eating habits, and increased physical activity. The less healthy lifestyle behavior change group reported significant weight gain, deteriorated eating habits and sleep quality, unchanged/increased alcohol intake and smoking, and decreased physical activity. Among risk factors, body image dissatisfaction (odds ratio [OR] 8.8, 95% CI 5.3-14.7), depression (OR 1.8, 95% CI 1.3-2.5), increased stress level (OR 3.4, 95% CI 2.0-5.8), and gender minority identity (OR 5.5, 95% CI 1.3-22.3) were associated with adopting less healthy behaviors in adjusted models.

**Conclusions:**

The COVID-19 pandemic has appeared to have influenced lifestyle behaviors unfavorably in some but favorably in others. Body image perception, change in stress level, and gender identity are factors associated with behavior change patterns; whether these will sustain over time remains to be studied. Findings provide insights into developing strategies for supporting adults with poorer mental well-being in the postpandemic context and promoting healthful behaviors during future disease outbreaks.

**Trial Registration:**

ClinicalTrials.gov NCT04407533; https://clinicaltrials.gov/ct2/show/NCT04407533

## Introduction

On March 11, 2020, the World Health Organization (WHO) declared COVID-19 (SARS-CoV-2) a global pandemic. Between March 12 and 22, all Canadian provinces had declared a public health emergency, and individuals were asked to stay at home and nonessential businesses were closed, including restaurants and gyms. Indoor and outdoor gatherings were also prohibited in large provinces to avoid spreading of the disease. Indeed, the pandemic imposed a sudden disruption in people’s usual routine. Although many adopted telework practice, others, such as frontline workers, were required to work on-site with additional protective equipment. In May 2020, measures became less stringent and nonessential businesses across Canada reopened gradually [1]. COVID-19 vaccines in adults were under clinical trial investigation, when a second wave hit in September 2020, leading to the enforcement of a second lockdown across provinces [1,2].

Confinement inevitably disturbs lifestyle behaviors, such as eating habits, physical activity level, and sleep, to varying degrees among individuals [3]. Although some observational studies and surveys have reported more home cooking, better diet quality, and increased physical activity levels [4,5], others have observed decreased physical activity, increased energy and alcohol intake, binge eating, reliance on take-out foods, and overall decreased diet quality [6-8]. The combination of poor diet quality, low physical activity, and greater alcohol intake, BMI, and smoking represents an important risk for severe COVID-19 [9], COVID-19 mortality [10], and chronic diseases during the postpandemic period [11]. Accumulating evidence shows that particular groups, such as women and younger individuals, were more susceptible to higher levels of stress and depression during the pandemic, possibly due to changes in home life among others [12-15]. These factors, combined with limited access to grocery shops [8], restaurants, and food banks, restricted budgets due to sudden unemployment, and changes in living arrangements may have affected how people modified their lifestyle during the pandemic [16,17]. Yet, it remains unclear which factors are major determinants of overall lifestyle pattern adoption in times of the pandemic, and no study has examined the lifestyle pattern change of adults in Canada.

Therefore, the objectives of this study were to characterize the potential impact of the COVID-19 pandemic on the lifestyle habits of Canadians and to identify patterns of weight and lifestyle behavior change and associated risk factors. We postulated that individuals experiencing greater levels of stress, isolation, changes in their living situation and work arrangement, and decreased income adopted less healthy lifestyle behaviors. Findings from this study may inform strategies to cope with the effects of current and potential future outbreaks in promoting and maintaining a healthy lifestyle.

## Methods

### Study Population

The web- and mobile-based Canadian COVIDiet cohort study was initiated on May 29, 2020, toward the end of the COVID-19 pandemic’s first wave of infections. The primary objective was to provide longitudinal data on the impact of the pandemic on eating behaviors, diet quality, and related lifestyle behaviors of Canadian adults. Based on the Canadian population estimated at 31 million adults and considering a 5% margin of error, 95% CI, and 50% response distribution [18], 400 participants were required per age strata (18-45, 45-65, ≥65 years). Following power allocation relative to population size [19], the target sample size was 1920 participants. Until December 5, 2020, 2959 adults from across Canada were recruited using social media advertisement, professional societies, and institutional email lists and from community centers, including food banks. Efforts were made to recruit across age groups, regions, and gender (men and women). Regions were delimited as Ontario, Québec, British Columbia, Prairies (Alberta, Manitoba, Saskatchewan), Atlantic provinces (Nova Scotia, New Brunswick, Newfoundland and Labrador, Prince Edward Island), and territories (Northwest Territories, Yukon, and Nunavut). Interested individuals were invited to contact the study coordinator by email or visit the study website for more information and for eligibility screening.

Eligible participants were 18 years of age or older, were living in Canada at the time of study entry, were able to read English or French, owned a smartphone or tablet, and had access to the internet. Individuals living in care homes or other institutional environments; hospitalized, pregnant, or breastfeeding; or with an active and uncontrolled acute disease that interfered with usual food intake were excluded. The COVIDiet study participants were followed every 3 months for 1 year to capture the impact of the pandemic across waves of infections and for 1 year thereafter to evaluate the longer-term effect. The current analysis was conducted on baseline (May-December 2020) and retrospective data from the baseline questionnaire. Individuals who did not fill out the baseline questionnaire (n=904), provided implausible responses (n=8), and had missing data on changes in weight and lifestyle behavior (eating habits, alcohol consumption, physical activity level, sleep quality and smoking; n=442) were excluded. The final sample included 1609 participants (Multimedia Appendix 1, Figure S1).

### Ethical Considerations

Data were collected with online encrypted surveys using AirTable [20] and deidentified using generated ID codes. Participants were eligible to win a CA $250 (US $182) gift card when they enrolled in the study; 1 draw occurred at every 500 participants enrolled. All participants provided online informed consent. This study was approved by the McGill University Research Ethics Board (20-04-064) and was registered on ClinicalTrials.gov (NCT04407533).

### Questionnaires

#### Baseline Sociodemographics and Medical History

Participants were asked to provide demographic characteristics and changes in lifestyle habits since the pandemic. They were asked to report on age, self-identified gender (women, men, others), ethnicity (White, South Asian, Chinese, Black, Filipino, Latin American, Arabic, Southeast Asian, West Asian, Korean, Japanese, Indigenous, other), usual occupation (full-time, part-time, occasional/seasonal, self-employed, student, unemployed, caregiver, volunteer, retiree), highest or current level of education (grade 8 or lower, grade 9 or 10, high school diploma, vocational school, college diploma , university certificate, bachelor’s degree, graduate studies), usual annual household income (<CA $20,000/<US $14,560, CA $20,000-$50,000/US $14,560-$36,400, >CA $50,000 -$100,000/>US $36,400-$72,800, >CA $100,000-$150,000/>US $72,800-$109,199, >CA $150,000/>US $109,199), usual household/living situation (living with parents, grandparents, brothers and sisters, host family, roommates, partner, children, alone), province, area (urban, suburban, rural), height (m or ft), usual weight (kg or lb), perceived body image (satisfied, somewhat satisfied, not satisfied), living with a chronic disease (hypertension, osteoarthritis, mood and anxiety disorder, osteoporosis, diabetes, asthma, chronic obstructive pulmonary disease, ischemic heart disease, cancer, dementia, other), and time since diagnosis. Questions were retrieved from the Canadian Community Health Survey (CCHS) 2020 [21]. As a measure of restrictive public health measures burden, the pandemic wave at the time of study enrollment was used. Participants who completed the Rapid Response - Healthy Living questionnaire before the second wave was declared on September 23, 2020 (May 26-September 22, 2020) [22] were classified within the “lenient restrictions” category, while those who started the study after this cut-off (September 23-December 14, 2020) were classified within the “strict restrictions” category.

#### Other Lifestyle Behaviors

Participants were also asked to report on physical activity (≥ or <150 minutes/week [23]) and its intensity, based on the CCHS 2020 questionnaire. We queried on alcohol consumption (≤1 drink/week, 1-3 drinks/week, 4-8 drinks/week, 9-14 drinks/week, >15 drinks/week) using an adapted CCHS 2020 questionnaire to better capture short-term changes in alcohol intake since the start of the pandemic. Smoking (daily, occasionally, no) and sleeping time (hours) and quality (very good, fairly good, fairly bad, very bad) were also retrieved from the CCHS [24]. Participants answered according to the current situation and from before the pandemic. The current anxiety level was assessed using the General Anxiety Disorder-7 (GAD-7) [25], and depression was assessed using the 10-item Center for Epidemiologic Studies Depression Scale (CES-D-10; ≥10) [26]. We also queried on the self-reported change in the stress level compared to before the pandemic. If participants reported having 1 or more chronic diseases, questions were asked on medication and medication changes during the pandemic.

For each question, participants were given the option to respond with “I don’t know” or “I prefer not to answer.”

### Weight and Lifestyle Behavior Changes

Baseline (during) versus prepandemic changes in weight and lifestyle factor status were categorized into 3 levels. For weight, a difference greater than +/–3% was considered an increase or a decrease in weight, respectively; participants with 0%-2.9% or insignificant weight change were categorized as having unchanged weight. For alcohol consumption, an upward change in 1 or more categories (<1 drink/week, 1-3 drinks/week, 4-8 drinks/week, 9-14 drinks/week, ≥15 drinks/week) was categorized as increased consumption, a downward change as decreased consumption, or no change as unchanged consumption. For physical activity level, a change in adherence to the WHO recommendations (yes or no) was categorized as increased (no to yes), decreased (yes to no), or unchanged (yes to yes or no to no). For eating habits, a perceived change was reported as improved, unchanged, or worsened. For sleep quality, an increase or decrease in 1 or more categories (very bad, fairly bad, fairly good, very good) was categorized as increased or decreased sleep quality, respectively, or otherwise categorized as unchanged. For smoking status (daily or occasionally/never), a change was categorized as increased (no to yes), decreased (yes to no), or unchanged (yes to yes, or no to no).

#### Statistical Analysis

Baseline characteristics were reported as means and SDs, and counts and percentages, as applicable. Latent class analysis (LCA) was used to identify lifestyle behavior change patterns based on 6 indicator variables: change in weight, alcohol consumption, physical activity, perceived eating habits, sleep quality, and smoking status. Models with 1, 2, and 3 classes were tested, and improvement in test statistics chi-square goodness of fit, Bayesian information criterion (BIC), Akaike information criterion (AIC), and entropy were used for model selection. Differences between participants’ characteristics and sociodemographics by class membership were examined using the chi-square for proportions, and the *t* test and Mann-Whitney U test for normally and nonnormally distributed data, respectively. Potential risk determinants of class membership were examined using logistic regressions. Model 1 was adjusted for age, gender, ethnicity, province, and burden of restrictive measure, and model 2 was adjusted for model 1 covariates and for body image perception, anxiety level, depression, chronic diseases, and changes in income, stress level, living situation, and work arrangement. Given the oversampling of women, gender-weighted sensitivity analysis was performed applying the Canadian 2019 Census gender proportions (women: 50.6%, men: 49.3%) [27] to improve the generalizability of results. Unweighted stratified analyses by age (< and ≥55 years), baseline BMI (< and ≥25 kg/m^2^), and restrictive public health measure burden subgroups were conducted. P<.05 was considered statistically significant in all analyses. LCA and logistic regressions were performed using R version 4.1.2 (2021-11) in RStudio (R Foundation for Statistical Computing). This study’s program is available on GitHub [28].

## Results

### Participants Characteristics

In total, 942 (46%) of 1609 participants were recruited between the end of the first wave of infections and the beginning of the second wave, that is, when lenient restrictive measures were in place. The other participants (1105/1609, 54%,) were recruited during the second wave of the pandemic, during strict restrictive measures. Table 1 shows prepandemic sociodemographic and lifestyle characteristics of participants. Participants mostly self-identified as women, were aged 18-89 years (mean 40.2, SD 15.5 years), and had a mean BMI of 26.1 (SD 6.3) kg/m^2^. The majority were White, had at least a bachelor’s degree, and reported having a household income > CA $50,000 (US $36,400) per year.

**Table 1:** Characteristics of participants, overall and by identified pattern of behavior change.

Characteristics		Overall (N=1609)	Healthy (n=974, 60.5%)	Less healthy (n=635, 39.5%)
**Gender, n (%); P<.001^a^**				
	Men	115 (7.1)	86 (8.8)	29 (4.6)
	Women	1461 (90.8)	873 (89.6)	588 (92.6)
	Minority	22 (1.4)	6 (0.6)	16 (2.5)
	Missing	11 (0.7)	9 (0.9)	2 (0.3)
**Age (years) at baseline, mean (SD); range=18-89 years, P=.26^b^**		40.2 (15.5)	40.5 (16.1)	39.6 (14.3)
	Missing, n (%)^c^	20 (1.2)	N/A^d^	N/A
**Ethnicity, n (%); P<.001^a^**				
	White	1307 (81.2)	796 (81.7)	511 (80.5)
	Black	12 (0.7)	7 (0.7)	5 (0.8)
	Hispanic	31 (1.9)	18 (1.8)	13 (2.0)
	Asian	115 (7.1)	71 (7.3)	44 (6.9)
	Indigenous	11 (0.7)	3 (0.3)	8 (1.3)
	Multiethnic	106 (6.6)	63 (6.5)	43 (6.8)
	Missing	27 (1.7)	16 (1.6)	11 (1.7)
**Province or territory of residence, n (%); P<.001^a^**				
	Pacific region	186 (11.6)	106 (10.9)	80 (12.6)
	Prairie provinces	310 (19.3)	173 (17.8)	137 (21.6)
	Ontario	603 (37.5)	342 (35.1)	261 (41.1)
	Québec	372 (23.1)	273 (28.0)	99 (15.6)
	Atlantic region	120 (7.5)	67 (6.9)	53 (8.3)
	Northern Territories	7 (0.4)	6 (0.6)	1 (0.2)
	Missing	11 (0.7)	7 (0.7)	4 (0.6)
**Residential area, n (%); P=.12^a^**				
	Urban	912 (56.7)	549 (56.4)	363 (57.2)
	Suburban	445 (27.7)	284 (29.2)	161 (25.4)
	Rural	247 (15.4)	138 (14.2)	109 (17.2)
	Missing	5 (0.3)	3 (0.3)	2 (0.3)
**BMI (kg/m^2^), mean (SD); P<.001^b^**		26.1 (6.3)	25.7 (6.2)	26.8 (6.5)
	Missing, n (%)	77 (4.8)	N/A	N/A
**Education, n (%); P=.96^a^**				
	Grade 9 or 10	5 (0.3)	4 (0.4)	1 (0.2)
	High school diploma	197 (12.2)	116 (11.9)	81 (12.8)
	Vocational school	14 (0.9)	8 (0.8)	6 (0.9)
	Diploma from Collège d'enseignement general et professionnel (CEGEP) or community college	279 (17.3)	167 (17.1)	112 (17.6)
	University certificate	91 (5.7)	54 (5.5)	37 (5.8)
	Bachelor’s degree	599 (37.2)	370 (38.0)	229 (36.1)
	Graduate studies (master’s degree or PhD)	381 (23.7)	231 (23.7)	150 (23.6)
	Missing	43 (2.7)	24 (2.6)	19 (3.0)
**Household income (CA $/US $ per year)^e^, n (%); P=.08^a^**				
	<20,000/<14,560	110 (6.8)	59 (6.1)	51 (8.0)
	20,000-50,000/14,560-36,400	315 (19.6)	181 (18.6)	134 (21.1)
	>50,000-100,000/>36,400-72,800	432 (26.8)	261 (26.8)	171 (26.9)
	>100,000-150,000/>72,800-109,199	335 (20.8)	199 (20.4)	136 (21.4)
	>150,000/>109,199	269 (16.7)	183 (18.8)	86 (13.5)
	Missing	148 (9.2)	91 (9.3)	57 (9.0)
**Employment status, n (%); P=.32^a^**				
	Full-time	734 (45.6)	436 (44.8)	298 (46.9)
	Part-time	176 (10.9)	109 (11.2)	67 (10.6)
	Student	103 (6.4)	63 (6.5)	40 (6.3)
	Retired	145 (9.0)	99 (10.2)	46 (7.2)
	Other (caregiver, volunteer, etc)	440 (27.3)	259 (26.6)	181 (28.5)
	Missing	11 (0.7)	8 (0.8)	3 (0.5)
**Chronic diseases, n (%); P=.08^a^**				
	0	1041 (64.7)	633 (65.0)	408 (64.3)
	1	142 (8.8)	87 (8.9)	55 (8.7)
	≥2	241 (15.0)	131 (13.4)	110 (17.3)
	Missing	185 (11.5)	123 (12.6)	62 (9.8)
**Smoker, n (%)**		117 (7.3)	71 (7.3)	46 (7.2)
**Alcohol consumption, n (%); P<.001^a^**				
	≥4 drinks/week	366 (22.7)	258 (26.5)	108 (17.0)
**Restrictive measures at enrollment, n (%); P<.001^a^**				
	Strict	1033 (64.2)	584 (60.0)	449 (70.7)
	Lenient	576 (35.8)	390 (40.0)	186 (29.3)
**Use of dietary supplements, n (%); P=.02^a^**				
	Yes	596 (37.0)	340 (34.9)	256 (40.3)
	No	999 (62.1)	627 (64.4)	372 (58.6)
	Missing	14 (0.9)	7 (0.7)	7 (1.1)
**Physical activity, n (%); P<.001^a^**				
	<150 minutes/week	592 (36.8)	581 (59.7)	436 (68.7)
	≥150 minutes/week	1017 (63.2)	393 (40.3)	199 (31.3)
**Since the pandemic, mean (SD)**				
	GAD-7^f^, anxiety symptoms (/21); P<.001^b^	7.1 (5.3)	6.1 (4.8)	8.8 (5.5)
	CES-D-10^g^, depression symptoms (/30); P<.001^b^	11.1 (6.7)	9.3 (6.1)	13.7 (6.6)
**Body image perception, n (%); P<.001^a^**				
	Satisfied	235 (14.6)	205 (21.0)	30 (4.7)
	Somewhat satisfied	655 (40.7)	453 (46.5)	202 (31.8)
	Not satisfied	707 (43.9)	306 (31.4)	401 (63.1)
	Missing	12 (0.7)	10 (1.0)	2 (0.3)

^a^P values are from chi-square tests unless otherwise indicated.

^b^P values are from 2-sided independent *t* tests.

^c^All percentages are unweighted.

^d^N/A: not applicable.

^e^CA $1.00=US $0.73.

^f^GAD-7: General Anxiety Disorder-7

^g^CES-D-10: 10-item Center for Epidemiologic Studies Depression Scale.

### Self-Reported Changes in Weight, Lifestyle, and Living Factors

Since the pandemic, 35.1% (565/1609) participants had a decreased income, 49.0% (788/1609) changed their work arrangement, and 94.3% (1518/1609) kept the same living situation (1295/1609, 80.5%, were living with others, and 223/1609, 13.9%, were living alone). Regarding mental well-being, 52.2% (840/1609) participants reported a worsened mood and 63.8% (1026/1609) reported increased stress levels compared to before the pandemic. Among lifestyle factors, most of the population reported either no change or insignificant weight gain or loss and no change in alcohol intake, physical activity, smoking, and sleep quality, yet 44.1% (709/1609) reported a decreased perceived quality of eating habits (Table 2).

**Table 2:** Self-reported changes in sociodemographic status, weight, and lifestyle factors. All reported values are unweighted.

Factor	Increased, n (%)	Unchanged, n (%)	Decreased, n (%)
Weight	523 (32.5)	920 (57.2)	166 (10.3)
Perceived eating habit quality	369 (22.9)	531 (33.0)	709 (44.1)
Sleep quality	219 (13.6)	815 (50.7)	575 (35.7)
Physical activity level	196 (12.2)	1001 (62.2)	412 (25.6)
Smoking	17 (1.1)	1559 (96.9)	33 (2.1)
Alcohol consumption	412 (25.6)	991 (61.6)	206 (12.8)

### Emergent Patterns of Change in Weight and Lifestyle Behaviors

**Figure 1:**
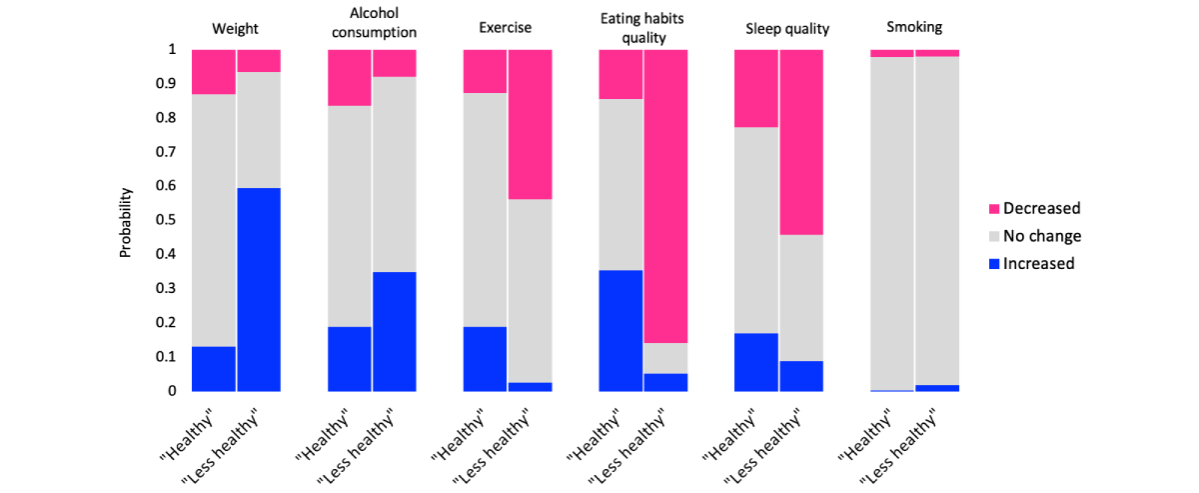
Two identified classes of change in weight and lifestyle patterns as a result of the pandemic. Classes were identified using LCA based on self-reported changes (prepandemic vs baseline) in weight, physical activity level, smoking, alcohol use, and sleep quality. LCA: latent class analysis.

Among the tested LCA models, the model with 2 emerging lifestyle behavior change classes had the best fit despite a slightly lower AIC compared to the model with 3 classes (Multimedia Appendix 2, Table S1) and was therefore selected for use. Participants with the class 1 membership (974/1609, 60.5%, population share probability) were more likely to report unchanged weight, sleep quality, smoking and alcohol intake, unchanged/improved eating habits, and increased physical activity. Participants with the class 2 membership (635/1609, 39.5%, population share probability) were more likely to report significant weight gain, deteriorated eating habits and sleep quality, unchanged/increased alcohol intake and smoking, and decreased physical activity. Class 1 was referred to as the healthy and class 2 as the less healthy pattern. Results from the LCA are presented in Figure 1.

Participants with the less healthy pattern were more likely to self-identify as women or a gender minority, to have a lower income prepandemic, and to live with 2 or more chronic diseases. They were also more likely to report a decreased income since the pandemic. Age, ethnicity, residential area, and change in the living situation did not differ between classes.

### Associations of Risk Factors With Identified Patterns of Lifestyle Behavior Change

**Figure 2:**
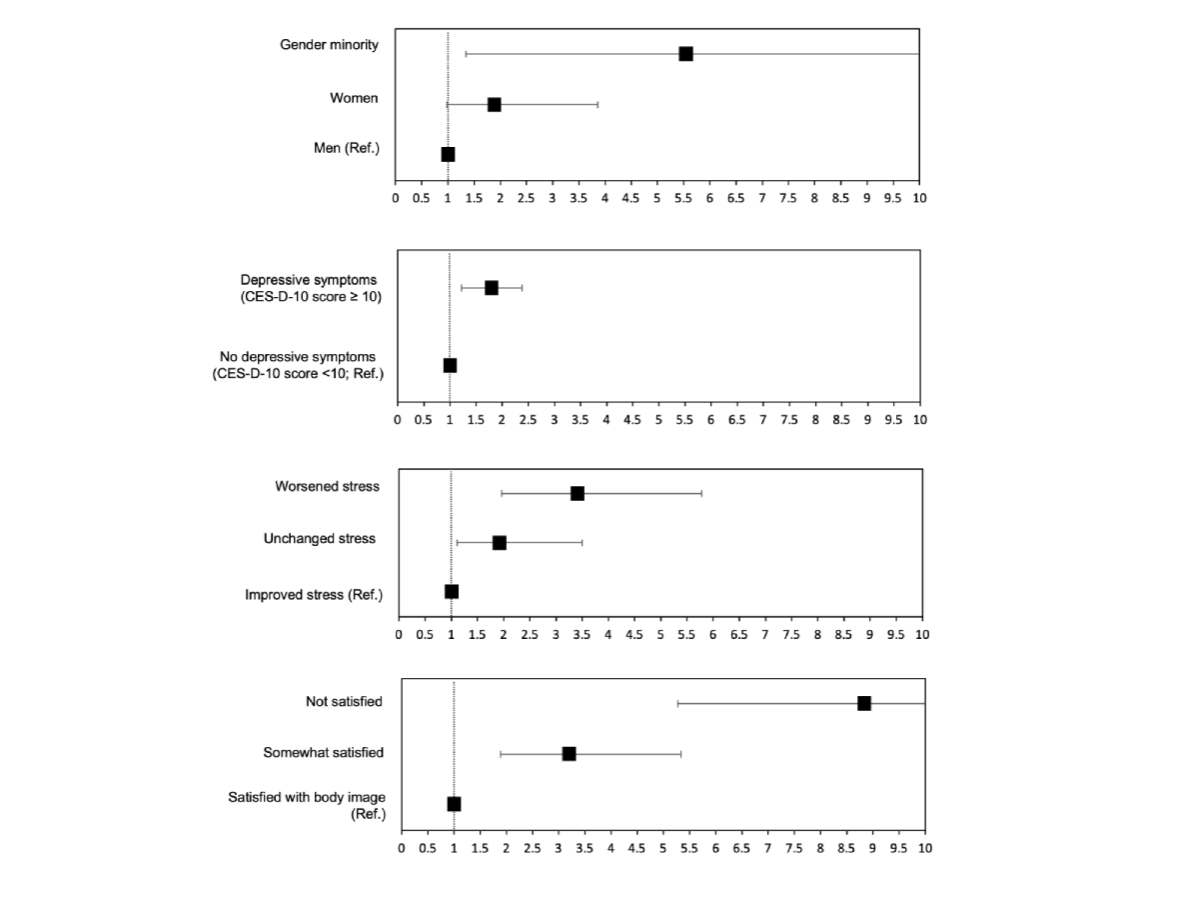
Unweighted adjusted ORs of the association between psychosocial factors and lifestyle pattern adoption (“less healthy” vs "healthy”). CES-D-10, 10-item Center for Epidemiologic Studies Depression Scale; OR, odds ratio; Ref., reference group. Tables S2 and S3 provide unweighted and gender-weighted ORs.

Among risk factors, body image dissatisfaction (odds ratio [OR] 8.8, 95% CI 5.3-14.9), increased stress level (OR 3.4, 95% CI 2.0-5.8), depression (OR 1.7, 95% CI 1.2-2.4), and self-identifying as a gender minority (OR 5.5, 95% CI 1.3-22.3) were positively associated with adopting less healthy lifestyle behaviors in adjusted models (Figure 2 and Multimedia Appendix 2, Table S2). In addition, the province of Québec was inversely associated with less healthy lifestyle behavior changes (OR 0.51, 95% CI 0.33-0.80).

Gender-weighted sensitivity analysis showed a stronger positive association between women and the less healthy lifestyle behavior changes, and the association for gender minorities disappeared (Multimedia Appendix 2, Table S3). The results were otherwise consistent with the nonweighted analysis.

Stratified analysis by age group showed positive associations of body image dissatisfaction, symptoms of depression, increased stress, and women and minority genders with less healthy lifestyle behavior changes in younger adults (<55 years old) and positive associations of symptoms of anxiety and body image dissatisfaction with less healthy lifestyle behavior changes in older adults (Multimedia Appendix 2, Table S4). However, depressive symptoms, gender, and stress level were not determinants of lifestyle behavior change patterns in this subgroup. Living with a chronic disease was predictive of less healthy lifestyle behavior changes in older adults.

Stratified analysis by BMI group showed positive associations between body image dissatisfaction, symptoms of depression, increased stress levels, lenient restrictive measures at the time of enrollment, living with ≥2 chronic diseases, living situation change (lived with people and moved alone), and less healthy lifestyle behavior changes in those with a usual BMI of <25 kg/m^2^ (Multimedia Appendix 2, Table S5). In participants with a usual BMI of ≥25 kg/m^2^, increased stress levels, maintenance of the same work arrangement, and body image dissatisfaction were positively associated with less healthy lifestyle behavior changes. Living with ≥2 chronic diseases was predictive of less healthy lifestyle behavior changes in this population subgroup. Gender was not a determinant of the outcome within either BMI group.

Stratified analysis by the burden of restrictive measures at the time of enrollment showed body image dissatisfaction, symptoms of depression, and increased levels of stress to be positively associated with less heathy lifestyle behavior changes in both subgroups (Multimedia Appendix 2, Table S6). Living with ≥2 chronic diseases during lenient restrictive measures was predictive of less healthy lifestyle behavior changes. None of the participants recruited during lenient restrictive measures self-identified as a gender minority; gender was only significant within the subgroup of participants recruited during strict restrictive measures.

## Discussion

### Principal Findings

During the COVID-19 pandemic, more than half of the Canadian COVIDiet study adults reported no change in body weight, smoking status, exercise level, alcohol intake, and sleep quality; however, a dominant proportion (44%) reported worsened eating habits. Considering these lifestyle factors altogether, we identified 2 distinct lifestyle behavior change patterns: healthy and less healthy. The significant and independent determinants of a less healthy lifestyle behavior change pattern were body dissatisfaction, depression, increased stress levels since the pandemic, self-identifying as a gender minority, and the province of residence.

Several surveys have reported on changes in individual lifestyle factors during the pandemic in adults worldwide [10,29-40], but few observational studies have examined the overall lifestyle behavior change patterns and associated risk factors [41,42]. Similar to our findings and using comparable data-driven statistical approaches, one study found “favorable,” “unfavorable,” and “no change” lifestyle change groups in a French cohort [42], while another study identified “prohealthy,” “unhealthy,” and “constant” groups in a Polish population [41].

### Adopting Healthy Lifestyle Behaviors

In this study, the healthy lifestyle behavior change pattern (974/1609, 60.5%) was mainly characterized by an increased physical activity level, unchanged or improved perceived eating habits, and stable weight, sleep quality, and smoking status. An increased physical activity level has also been reported in other countries [29,42]. It may have resulted from increased free time due to telework or from a desire to improve personal health in facing the disease outbreak. In addition, participants allocated to the healthy class were more active at baseline, which may have led them to coping with the pandemic-related stress with exercise [43], thus increasing their physical activity level, while decreasing stress levels. With regard to diet, stable and improved eating habits were observed in our study and elsewhere [4,29,42]. Staying at home may have favored the avoidance of less healthy food, such as fast food, and more frequent cooking for some individuals [4,29,42,44]. One study in the Canadian province of Québec (NutriQuebec study; N=853, with 87.2% women, 52.5% aged 50-69 years) showed a slight overall increase in diet quality (+1.1/100 points in the Healthy Eating Index-2015; 95% CI 0.6-1.5) during early lockdown (April-May 2020), especially in younger adults, in those with lower education, or in those with obesity [4]. Although not directly comparable, only 22.9% (369/1609) of the overall population reported improved eating habits and a larger proportion reported worsened eating habits (709/1609, 44.1%) in our study. In addition, only 35.5% (346/974) reported improved eating habits and the majority reported unchanged habits in the healthy lifestyle behavior change group. This suggests that the diet quality of Canadians may not have improved at a population level; in-depth dietary assessment will permit more robust conclusions. Lastly, the COVID-19 pandemic appears to have had disproportionate impacts on populations living with social inequalities [45]. Participants in the healthy pattern group were more likely to self-identify as men, to report higher and unchanged household incomes, to be satisfied with their body image, and to be living with less than 2 chronic diseases. The lifestyle behavior changes observed in this group may be reflective of social privilege.

### Adopting Less Healthy Lifestyle Behaviors

Contrastingly, the less healthy lifestyle behavior change pattern (635/1609, 39.5%) was characterized by perceived worsened eating habits, decreased physical activity, decreased sleep quality, and weight gain. Similar patterns of lifestyle habit deterioration were observed in Canada, predominantly in British Columbia [46], and in other countries [47]. Another Canadian study (N=2338, with 90.2% women, February-April 2020) showed that although moderate-to-vigorous activity tracked with wearable devices returned to the prepandemic level 6 weeks after an initial decline in March 2020, the step count and light physical activity remained lower [48]. The lockdown imposed important modifications on daily physical activities, including work-related commuting interruption due to telework or unemployment, the prohibited practice of team sports, and the closure of fitness centers. Those at higher risk of complications and particularly worried of contracting COVID-19 possibly took extra isolation precautions and reduced their physical activity level. In addition, our data show that 1 independent determinant of the less healthy classification was increased levels of stress. Both objective and subjective measures of stress have been shown to hinder physical activity levels, especially in less active individuals [43]. Worsened eating habits may be explained by modified food supply and food choices due to more limited access to grocery stores, fresh food, and working from home. Increased snacking and sweet consumption have been reported in numerous global studies (reviewed in [49]) and may have contributed to participants’ perception of worsened eating habits. In addition, the less healthy lifestyle behavior change class more likely reported increased alcohol intake. Altogether, these factors probably explain the observed body weight increase in this group. Comparably, in the NutriNet-Santé cohort study (France; N=37,252; weighted proportions: 52.3% women, 42.5% aged 25-50 years), among the 3 clusters identified, 1 reflected unfavorable changes that included decreased physical activity, increased snacking and sweet consumption, decreased vegetable and fruit consumption, and weight gain between March and May 2020 [50]. These changes were associated with being a woman, working from home, the presence of children at home, a lower income, and more depressive symptoms. In our study, individuals in the less healthy lifestyle behavior change group also more likely reported increased stress levels and anxiety and depressive symptoms and a greater proportion had more than 2 chronic diseases and reported a decreased income. A healthy lifestyle, including regular physical activity and high diet quality, were associated with reduced risk of chronic diseases [51] and mortality [52]. As a strategy to promote health during self-quarantine, the WHO regional office for Europe released physical activity and dietary guidelines [52]. However, none were made available in Canada in the early months of the pandemic. The impact of such guidelines on public health during the pandemic remains to be assessed.

### Factors Associated With Lifestyle Behavior Changes

Among potential risk factors, body image satisfaction was the strongest determinant of lifestyle behavior change patterns during the pandemic, independent of several other factors, including gender, age, and income. Close to half of the studied population (707/1609, 43.9%) reported being not satisfied with their body image during the pandemic, a proportion higher than that observed in a population survey in Canada before the pandemic (15%) [53]. Body image dissatisfaction was also more frequent in those self-identifying as women and gender minorities, compared to men, which is typically observed in Western societies [54,55]. Evidence supports that individuals dissatisfied with their body image may have poorer mental health [56] and may engage in less healthy lifestyle behaviors, such as decreased physical activity [57]. A study showed an association of worsened body image dissatisfaction with increased psychological distress during the pandemic [58]. Yet, in our study, body image dissatisfaction was associated with less healthy lifestyle behavior changes independent of stress, anxiety, and depression. The reverse association is also possible, that is, those who adopted less healthy lifestyle behavior changes, including weight gain, may have experienced a greater level of dissatisfaction toward their body image. In our study, the majority of participants also reported increased stress levels since the pandemic (1026/1609, 63.8%), which was associated with less healthy lifestyle behavior changes. Compelling prepandemic evidence has supported an association of stress, anxiety, and depression with poorer lifestyle behaviors, including weight gain [59-63]. However, the pandemic appears to have exacerbated negative mental health in some individuals, putting them at risk of adopting poorer lifestyle behaviors [17] or vice versa [64].

Limited data of the impact of the COVID-19 pandemic on gender minorities have been collected and are available. In our study, self-identifying as a gender minority was associated with adopting less healthy lifestyle habits; although the sample size was small for this group (24/1609, 1.2%), the proportion was comparable to that reported in Canada (0.33% from Census data) [65]. Current evidence supports high rates of worsening physical and mental health outcomes during the pandemic among individuals belonging to gender minorities [66,67]. From stratified analyses, both self-identifying as a woman and self-identifying as a gender minority were associated with less healthy lifestyle behavior changes among younger participants (<55 years) only. Before the pandemic, women (compared to men) and gender-diverse individuals (compared to men and women) already reported poorer mental health in Canada. Since the beginning of social distancing, negative mental health has been reported in women and gender minorities in Canada [68] and elsewhere [58,69-71]. In people belonging to gender minorities, the lack or loss of access to community groups, stigmatization, and negative psychological outcomes of the pandemic may have contributed to the adoption of less healthy lifestyle behavior changes [66]. In younger women and gender minorities, the telework and at-home childcare arrangement may have further adversely impacted lifestyle behaviors [42,71].

Living in the province of Québec was significantly associated with adopting healthier lifestyle behaviors during the pandemic compared to Ontario and other large provinces. Yet, restrictive measures have been comparable across these provinces throughout the pandemic [1]. Some evidence suggests that the Québec population may have experienced healthier changes [4]. However, the association with healthier lifestyle behavior changes during the pandemic is most likely attributable to greater recruitment in this province during less stringent restrictive measures in our study (Québec: 301/576 during lenient and 71/1033 during strict restrictions; Ontario: 139/576 during lenient and 464/1033 during strict restrictions).

### Strengths and Limitations

The strengths of this study include the overall large sample size and adequate representation across provinces. Another strength is the use of LCA, as opposed to using measures of central tendency, that allowed us to identify 2 classes and informed on distinct patterns within the population.

The study also has limitations. First, variations in restriction measures were observed across provinces between the start and the end of recruitment, which may have contributed to confounding residuals, although our models were adjusted for the pandemic phases during which participants responded to the questionnaire. Second, the use of self-reported lifestyle questionnaires may have introduced subjectivity to measurements. Third, the use of advertisement strategies combined with the nature of our study may have led to selection bias of more educated and healthier individuals. In addition, the over-/undersampling of specific groups of individuals, namely gender and age, may have limited the generalizability of our results to the Canadian population; however, our sensitivity analysis using gender-based calibration weights showed consistent results with our main analysis (Multimedia Appendix 2, Table S3). Fourth, although subgroup analysis by age, baseline BMI, and pandemic wave group was successfully conducted, it was not possible to examine the associations between risk factors and lifestyle behavior change patterns across ethnic and gender groups; further studies with a larger sample size within each, especially ethnic and gender minorities, are warranted.

### Conclusion

Findings from this observational study showed that 39.5% (635/1609) of participants adopted less healthy lifestyle behaviors during the COVID-19 pandemic but that the majority (974/1609, 60.5%) adopted healthier behaviors. Factors such as body image perception, depression status, change in the stress level, and gender identity may have motivated these lifestyle behavior changes. It is yet to be determined whether the latter will sustain in the long term. In the postpandemic period, multidisciplinary strategies involving dietitians, psychologists, and kinesiologists may be considered to assist adults in (re)gaining mental and physical health. Lockdown-adapted public health recommendations and resources related to nutrition, physical activity, sleep, and smoking should be promoted during future outbreaks.

## Appendix

Multimedia Appendix 1 STROBE diagram showing the flow of participants in the Canadian COVIDiet cohort study. Inclusion criteria included reading and speaking English or French, owning a smartphone or tablet, and access to the internet. Exclusion criteria were living with an active and uncontrolled acute disease that interfered with usual food intake, being hospitalized, being pregnant, or living in care homes or other institutional environments.

Multimedia Appendix 2 Supplemental Tables 1-6.

## Abbreviations

AIC: Akaike information criterion
BIC: Bayesian information criterion
CCHS: Canadian Community Health Survey
CES-D-10: 10-item Center for Epidemiologic Studies Depression Scale
GAD-7: General Anxiety Disorder-7
LCA: latent class analysis
OR: odds ratio
WHO: World Health Organization

## Data Availability

The data sets generated and analyzed during the study are not publicly available due to ethical restrictions but are available from the corresponding author upon reasonable request.
