# Supplementary material for: Lifestyle Behavior Changes and Associated Risk Factors During the COVID-19 Pandemic: Results from the Canadian COVIDiet Online Cohort Study
Source: JMIR Public Health Surveill. 2023 Mar 30;9:e43786. doi: 10.2196/43786 (PMC10131911; doi:10.2196/43786)
Supplement: Multimedia Appendix 1 [file publichealth_v9i1e43786_app1.docx]

Multimedia Appendix 1


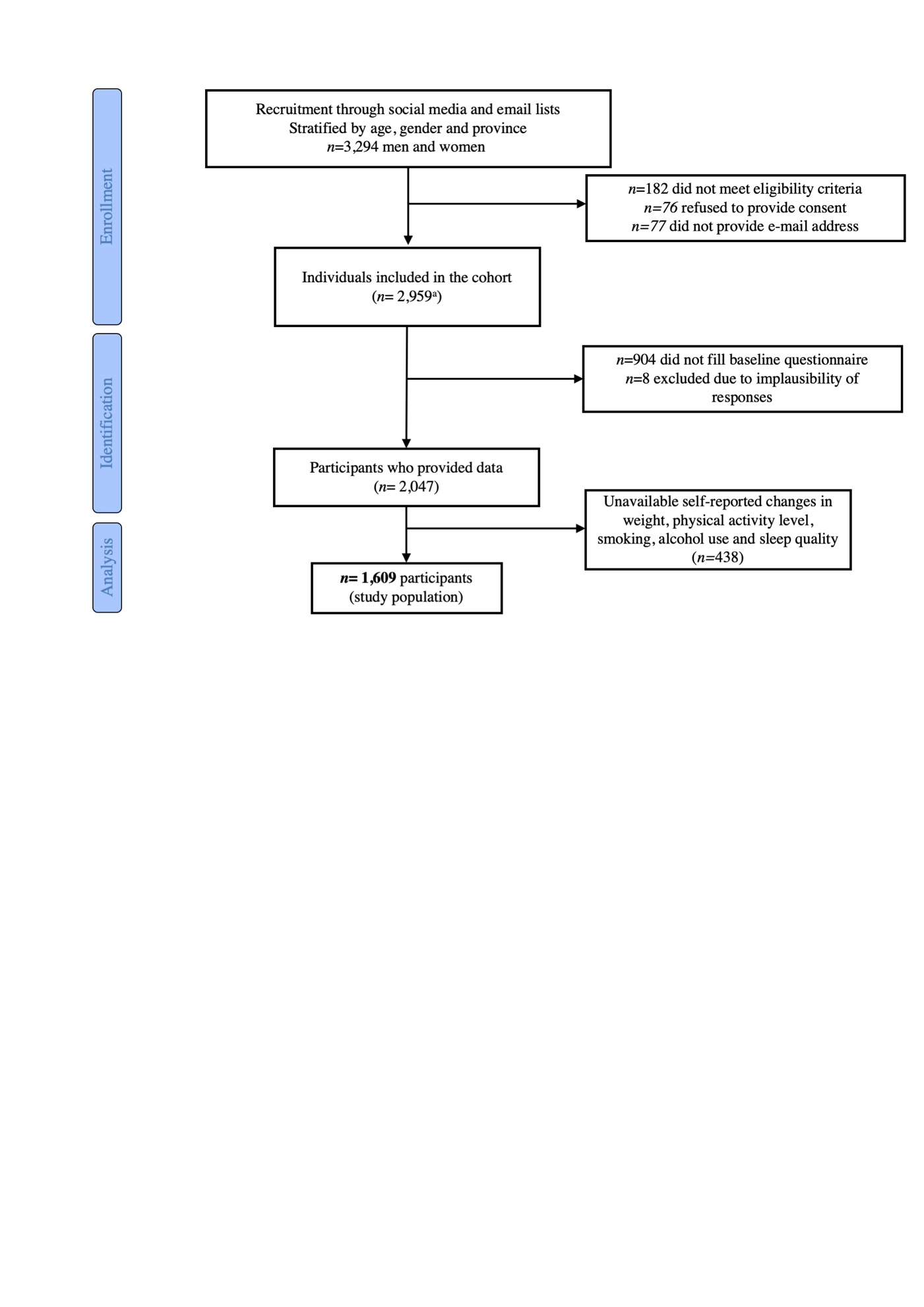
Figure S1 STROBE diagram showing the flow of participants in the Canadian COVIDiet cohort study. Inclusion criteria included reading and speaking English or French, owning a smartphone or tablet, and access to the internet. Exclusion criteria were living with an active and uncontrolled acute disease that interfered with usual food intake, being hospitalized, being pregnant, or living in care homes or other institutional environments.
