# Supplementary material for: Lifestyle Behavior Changes and Associated Risk Factors During the COVID-19 Pandemic: Results from the Canadian COVIDiet Online Cohort Study
Source: JMIR Public Health Surveill. 2023 Mar 30;9:e43786. doi: 10.2196/43786 (PMC10131911; doi:10.2196/43786)
Supplement: Multimedia Appendix 2 [file publichealth_v9i1e43786_app2.docx]

**Supplemental Tables**

Table S1. Test statistics of tested latent class analysis models with one, two and three classes

|  | One class | Two classes | Three classes |
| --- | --- | --- | --- |
| Residual degrees of freedom | 716 | 703 | 690 |
| Chi-square goodness of fit | 2625.8 | 1633.3 | 982.7 |
| Likelihood ratio/deviance statistic | 1116.8 | 619.0 | 448.2 |
| AIC | 15911.5 | 15439.7 | 15294.8 |
| BIC | 15976.1 | 15574.3 | 15499.4 |
| Entropy | 4.94 | 4.78 | 4.73 |

AIC, Akaike information criterion, BIC, Bayesian information criterion.

Table S2. Unweighted associations between potential risk factors and “less healthy” lifestyle behaviour change

|  | Model 1 | | Model 2 | |
| --- | --- | --- | --- | --- |
|  | OR (95%CI) | *P* value | OR (95% CI) | *P* value |
| Women  (Ref. men) | 2.48 (1.35, 4.57) | .004 | 1.95 (0.98, 3.86) | .055 |
| Gender minority  (Ref. men) | 9.60 (2.64, 34.90) | <.001 | 5.47 (1.34, 22.32) | .02 |
| Province of Quebec  (Ref. Ontario) | 0.48 (0.33, 0.70) | <.001 | 0.51 (0.33, 0.80) | .003 |
| Lenient restrictive measures (Ref. strict measures) |  |  | 0.70 (0.49, 0.99) | .046 |
| Body image - somewhat satisfied  (Ref. satisfied) | - | - | 3.18 (1.89, 5.33) | <.001 |
| Body image – not satisfied  (Ref. satisfied) | - | - | 8.85 (5.27, 14.87) | <.001 |
| CES-D-10 score ≥ 10  (Ref. < 10) | - | - | 1.71 (1.22, 2.38) | .002 |
| Unchanged stress (Ref. improved) | - | - | 1.96 (1.11, 3.50) | .02 |
| Worsened stress (Ref. improved) |  | - | 3.37 (1.96, 5.78) | <.001 |
| Changed work arrangement (Ref. unchanged) | - | - | 1.35 (1.00, 1.82) | .050 |

OR, odds ratios; CI, confidence interval; Ref., reference group

Model 1: includes age, gender, ethnicity, province, stringency of public health restriction measures at enrollment

Model 2: includes model 1 factors + body image perception, chronic diseases, GAD-7, CES-D-10, change in stress level, income, living situation and work arrangement.

Table S3. Gender-weighted associations between potential risk factors and “less healthy” lifestyle behaviour change

|  | Model 1 | | Model 2 | |
| --- | --- | --- | --- | --- |
|  | OR (95%CI) | *P* value | OR (95% CI) | *P* value |
| Women  (Ref. men) | 2.35 (1.76, 3.13) | <.001 | 1.82 (1.27, 2.61) | .001 |
| Province of Quebec  (Ref. Ontario) | 0.50 (0.34, 0.76) | <.001 | 0.47 (0.27, 0.73) | .001 |
| Lenient restrictive measures (Ref. strict measures) |  |  | 0.84 (0.56, 1.26) | .046 |
| Body image - somewhat satisfied  (Ref. satisfied) | - | - | 3.98 (2.01, 7.88) | <.001 |
| Body image – not satisfied  (Ref. satisfied) | - | - | 16.2 (8.20, 32.02) | <.001 |
| CES-D-10 score ≥ 10  (Ref. < 10) | - | - | 1.90 (1.29, 2.81) | .001 |
| Unchanged stress (Ref. improved) | - | - | 2.40 (1.11, 5.20) | .03 |
| Worsened stress (Ref. improved) |  |  | 4.45 (2.12, 9.34) | <.001 |
| Changed work arrangement (Ref. unchanged) | - | - | 1.60 (1.12, 2.29) | .009 |

OR, odds ratios; CI, confidence interval; Ref., reference group

Model 1: includes for age, gender, ethnicity, province, stringency of public health restriction measures at enrollment

Model 2: includes model 1 factors + body image perception, chronic diseases, GAD-7, CES-D-10, change in stress level, income, living situation and work arrangement.

Gender minority is not reported in this table due to the small sample size attribution following weighing.

Analyses are weighted for gender as per the Canadian Census 2019 population proportions [26].

Table S4. Stratified analysis by age groups

|  | < 55 y  (n=845) | | >= 55 y  (n=379) | |
| --- | --- | --- | --- | --- |
|  | OR (95%CI) | *P* value | OR (95%CI) | *P* value |
| Women  (Ref. men) | 2.27 (0.99,5.21) | .05 | 1.56(0.43, 5.61) | .50 |
| Gender minority  (Ref. men) | 5.97 (1.35, 26.35) | .018 | - | - |
| Province of Quebec  (Ref. Ontario) | 0.45 (0.27, 0.75) | .002 | 0.90 (0.30, 2.72) | .85 |
| Lenient restrictive measures (Ref. strict measures) | 0.68 (0.45, 1.03) | .07 | 0.76 (0.35, 1.65) | .48 |
| Body image - somewhat satisfied  (Ref. satisfied) | 3.03 (1.65, 5.55) | <.001 | 3.83 (1.29, 11.36) | .02 |
| Body image – not satisfied  (Ref. satisfied) | 9.44 (5.10, 17,46) | <.001 | 9.61 (3.32, 27,85) | <.001 |
| CES-D-10 score ≥ 10  (Ref. < 10) | 1.84 (1.22, 2.80) | .004 | 1.32 (0.68, 2.58) | .41 |
| Unchanged stress (Ref. improved) | 2.77 (1.39, 5.54) | .004 | 0.39 (0.12, 1.30) | .12 |
| Worsened stress (Ref. improved) | 5.10 (2.66, 9.77) | <.001 | 0.52 (0.16, 1.68) | .28 |
| Changed work arrangement (Ref. unchanged) | 1.18 (0.83, 1.70) | 0.36 | 1.73 (0.94, 3.17) | .08 |

OR, odds ratios; CI, confidence interval; Ref., reference group; n=1224

Model 2, includes gender, ethnicity, province, stringency of public health restriction measures at enrollment, body image perception, chronic diseases, GAD-7, CES-D-10, change in stress level, income, living situation and work arrangement.

Analyses are unweighted.

Table S5. Stratified analysis by BMI groups

|  | < 25 kg/m2  (n=660) | | >= 25 kg/m2  (n=564) | |
| --- | --- | --- | --- | --- |
|  | OR (95%CI) | *P* value | OR (95%CI) | *P* value |
| Women  (Ref. men) | 3.94 (0.98, 15.76) | .05 | 1.39 (0.59, 3.26) | .45 |
| Gender minority  (Ref. men) | 8.03 (0.94, 68.74) | .06 | 11.70 (0.90, 151.91) | .06 |
| Province of Quebec  (Ref. Ontario) | 0.45 (0.25, 0.82) | .009 | 0.61 (0.30, 1.25) | .18 |
| Lenient restrictive measures (Ref. strict measures) | 0.52 (0.32, 0.84) | .007 | 1.00 (0.56, 1.78) | .99 |
| Body image somewhat satisfied  (Ref. satisfied) | 3.03 (1.67, 5.48) | <.001 | 4.72 (1.31, 16.93) | .02 |
| Body image dissatisfaction  (Ref. satisfied) | 9.70 (5.17, 18.21) | <.001 | 15.24 (4.30, 53.99) | <.001 |
| CES-D-10 score ≥ 10  (Ref. < 10) | 1.83 (1.13, 2.98) | .01 | 1.54 (0.93, 2.55) | .10 |
| Unchanged stress (Ref. improved) | 2.87 (1.25, 6.58) | .01 | 1.38 (0.57, 3.32) | .48 |
| Worsened stress (Ref. improved) | 3.97 (1.82, 8.66) | <.001 | 3.11 (1.36, 7.11) | .007 |
| Changed work arrangement (Ref. unchanged) | 1.03 (0.67, 1.59) | .90 | 1.73 (1.10, 2.71) | .02 |

OR, odds ratios; CI, confidence interval; Ref., reference group; n=1224

Model 2, includes age, gender, ethnicity, province, stringency of public health restriction measures at enrollment, body image perception, chronic diseases, GAD-7, CES-D-10, change in stress level, income, living situation and work arrangement.

Analyses are unweighted.

Table S6. Stratified analysis by stringency of public health restrictive measures at enrollment

|  | Lenient  (n=353) | | Strict  (n=871) | |
| --- | --- | --- | --- | --- |
|  | OR(95%CI) | *P* value | OR(95%CI) | *P* value |
| Women  (Ref.. men) | 3.59 (0.91, 14.17) | .07 | 1.56 (0.68, 3.60) | .29 |
| Gender minority  (Ref.. men) | - | - | 4.60 (1.05, 20.32) | .04 |
| Province of Quebec  (Ref.. Ontario) | 0.66 (0.33, 1.32) | .24 | 0.45 (0.22, 0.94) | .03 |
| Body image somewhat satisfied  (Ref. satisfied) | 3.55 (1.43, 8.87) | .007 | 3.35 (1.74, 6.46) | <.001 |
| Body image dissatisfaction  (Ref. satisfied) | 9.26 (3.50, 24.52) | <.001 | 9.88 (5.18, 18.83) | <.001 |
| CES-D-10 score ≥ 10  (Ref. < 10) | 2.11 (1.11, 4.02) | .02 | 1.70 (1.13, 2.57) | .01 |
| Unchanged stress (Ref. improved) | 2.94 (0.99, 8.77) | .05 | 1.70 (0.83, 3.50) | .14 |
| Worsened stress (Ref. improved) | 5.12 (1.77, 14.87) | .003 | 3.21 (1.67, 6.19) | <.001 |
| Changed work arrangement (Ref. unchanged) | 1.67 (0.92, 3.03) | .09 | 1.30 (0.90, 1.86) | .16 |

OR, odds ratios; CI, confidence interval; Ref., reference group; n=1224

Model 2, includes age, gender, ethnicity, province, body image perception, chronic diseases, GAD-7, CES-D-10, change in stress level, income, living situation and work arrangement.

Analyses are unweighted.

**References**

26. Statistics Canada. 2020. Sex at birth and gender: technical report on changes for the 2021 census [accessed 2023-03-14] <https://www12.statcan.gc.ca/census-recensement/2021/ref/98-20-0002/982000022020002-eng.cfm>
